# Hepatitis B virus in Pakistan: A systematic review of prevalence, risk factors, awareness status and genotypes

**DOI:** 10.1186/1743-422X-8-102

**Published:** 2011-03-06

**Authors:** Muhammad Ali, Muhammad Idrees, Liaqat Ali, Abrar Hussain, Irshad Ur Rehman, Sana Saleem, Samia Afzal, Sadia Butt

**Affiliations:** 1 Division of Molecular Virology, National Centre of Excellence in Molecular Biology, University of the Punjab, 87-West Canal Bank Road, Thokar Niaz Baig, Lahore, Pakistan

## Abstract

In Pakistan, there are estimated 7-9 million carriers of hepatitis B virus (HBV) with a carrier rate of 3-5%. This article reviews the available literature about the prevalence, risk factors, awareness status and genotypes of the HBV in Pakistan by using key words; HBV prevalence, risk factors, awareness status and genotypes in Pakistani population in PubMed, PakMediNet, Directory of Open Access Journals (DOAJ) and Google Scholar. One hundred and six different studies published from 1998 to 2010 were included in this study. Weighted mean and standard deviation were determined for each population group. The percentage of hepatitis B virus infection in general population was 4.3318% ± 1.644%, healthy blood donors (3.93% ± 1.58%), military recruits (4.276% ± 1.646%), healthcare persons (3.25% ± 1.202%), pregnant women (5.872% ± 4.984), prisoners (5.75% ± 0.212%), surgical patients (7.397% ± 2.012%), patients with cirrhosis (28.87% ± 11.90%), patients with HCC (22% ± 2.645%), patients with hepatitis (15.896% ± 14.824%), patients with liver diseases (27.54% ± 6.385%), multiple transfused patients (6.223% ± 2.121%), opthalmic patients (3.89% ± 1.004%) and users of injectable drugs (14.95% ± 10.536%). Genotype D (63.71%) is the most prevalent genotype in Pakistani population. Mass vaccination and awareness programs should be initiated on urgent basis especially in populations with HBV infection rates of more than 5%.

## Introduction

Hepatitis B virus (HBV) infection is a major global health problem [1-3], especially in Asia, Africa, southern Europe and Latin America [4]. About 2 billion people are infected with HBV worldwide [2,4,5], and 400 million among them are suffering from chronic HBV infection [6]. Pakistan is highly endemic with HBV [7] with nine million people infected with HBV [8] and its infection rate is on a steady rise [9]. The reason may be the lack of proper health facilities, poor economical status and less public awareness about the transmission of major communicable diseases including HBV, HCV and HIV [6].

The clinical course and sequel of chronic hepatitis vary among individuals. Infection with HBV leads to a wide spectrum of clinical presentations, ranging from asymptomatic carrier state to acute self-limiting infection or fulminant hepatic failure, chronic hepatitis with progression to cirrhosis, and hepatocellular carcinoma (HCC) [2].

Studies are too limited to give a clear picture of the prevalence of HBV at the National level, especially among otherwise healthy individuals. Most previous studies targeted different small groups of individuals with some clinical indications therefore; these do not accurately reflect the overall prevalence in Pakistan [7,8]. The present article briefly presents the prevalence, risk factors associated with HBV transmission, awareness status and HBV genotypes prevalent in Pakistani population.

## Literature Search and inclusion criteria

Articles were searched in PubMed, PakMediNet, Directory of Open Access Journals (DOAJ) and Google Scholar by using keywords; Hepatitis B virus in Pakistan, Prevalence of HBV in Pakistan, HBV in Blood donors, Hepatitis B virus in general population, HBV in Pakistani healthcare workers, HBV in surgical patients, HBV infection in women and children, HBV infection in prisoners, HBV in diseased population in Pakistan, HBV in injection drug users, epidemiology of HBV in Pakistan, HBV genotypes in Pakistan and awareness about HBV in Pakistani population. Two hundreds and twenty nine different studies (articles/repots) were obtained from the literature search, out of which 106 published from 1998 to 2010 were included in the present review. Studies full filling the following criteria were included: 1) Samples were collected from Pakistani individuals. 2) An obvious description of the methods of detection of HBV infection and genotyping. 3) Information about the number of individuals studied and their residing area were reported. 4) Studies reporting risk factors and awareness status in Pakistani population were included to discuss the HBV prevalence in different population groups.

## Analysis

Studies showing percent prevalence of HBV infection in different population are shown in table 1, 2, 3 and 4, while table 5 shows the percentage of different genotypes prevalent in Pakistan. The percent prevalence in the different population groups are presented in mean ± standard deviation (with 95% confidence interval).

**Table 1:** Prevalence of HBV in general population, young recruits and prisoners.

Population type	Region	Methods	Population size	HBV (%)	HBV marker	Reference
**General population**	Lahore	ELISA	992	8.06%	HBsAg	Nafees *et al*. [10] 2009
	Larkana	ICT, ELISA	200	4.8%	HBsAg	Shaikh *et al*. [11] 2009
	Lahore	ICT	203	2.46	HBsAg	Tanveer *et al*. [12] 2008
	Karachi	ICT, ELISA.	4000	4.5%	HBsAg	Noorali *et al*. [7] 2008
	Southern Punjab	ICT/ELISA	1821	5.9%	HBsAg	Mirza *et al*. [13] 2007
	Karachi	ICT, ELISA and PCR.	3820	4.5%	HBsAg & DNA	Hakim *et al*. [8] 2008
	Islamabad	AxSym HBsAg, CORE& AUSAB MEIA	1300	4%	HBsAg, anti HBs, anti HBc	Alam *et al*. [6] 2007
	Central Punjab	ICT, ELISA	2038	4.83%	HBsAg	Alam *et al*. [14] 2006
	Rawalpindi	ELISA	665	3%	HBsAg	Farooq *et al*. [15] 2005
	Lahore	ICT	757	2.6%	HBsAg	Amin *et al*. [16] 2004
	Karachi	ICT, ELISA	200	3%	HBsAg	Qasmi *et al*. [17] 2000
**Recruitments**	Rural Areas of Pakistan	ICT, ELISA	3320	4.5%	HBsAg	Azam *et al*. [18] 2009
	Interior Sindh	ICT, ELISA	5237	7.39%	HBsAg	Malik *et al*. [19] 2008
	Recruits from all over Pakistan	ICT, ELISA	2558	2.8%	HBsAg	Sherif & Tariq, [20] 2006
	Mardan	ICT, ELISA	15550	3.24%	HBsAg	Mirza *et al*. [21] 2006
	All areas of Pakistan	ICT, ELISA	4552	4.2%	HBsAg	Hussain *et al*. [22]2005
	All areas of Pakistan	ICT, ELISA	5371	3.53%	HBsAg	Ali *et al*. [23] 2002
**Prisoners**	Karachi	ELISA	365	5.9%	HBs Ag	Kazi *et al*. [24] 2010
	Bahawalpur	ICT, ELISA	2086	5.6%	HBs Ag	Fayyaz *et al*.[25] 2006

ELISA: Enzume linked immunosorbant assay; HBsAg: hepatitis B surface antigen; ICT: Immuno-chromatographic Test, MEIA: Microparticle Enzyme Immunoassay, HBsAg: hepatitis B surface antigen

**Table 2:** Percent prevalence rates of HBV in Healthy Blood donors.

Population type	Region	Methods	Population size	HBV positive (%)	HBV marker	Reference
**Healthy Blood Donors**	Kurram Agency	ICT	1300	5.07%	HBsAg	Bangash *et al*. [26] 2009
	Interior Sindh	ICT	5345	6.2%	HBsAg	Mujeeb & Pearce, [27] 2008
	Karachi	ICT	11459	1.71%	HBsAg	Nazar *et al*. [28] 2008
	Karachi	ICT	688	4.50%	HBsAg	Azam *et al*. [29] 2007
	Northern areas	ICT	8949	3.66%	HBsAg	Alam & Naeem, [30] 2007
	Karachi	ICT	21,125	3.3%	HBsAg	Mujeeb & Pearce, [31] 2007
	District Thatta	ICT	310	5.81%	HBsAg	Ishaq *et al*. [32] 2007
	Southern Punjab	ICT, ELISA	27938	2.69%	HBsAg	Khan *et al*. [33] 2006
	Lahore	ICT	18216	3.36%	HBsAg	Sirhindi *et al*. [34] 2005
	Karachi	ELISA	351309	2.0%	HBsAg	Akhtar *et al*. [35] 2005
	Peshawar/ KPK	MEIA	4000	1.9%	HBsAg	Ahmad *et al*. [36] 2004
	Rawalpindi	ICT	580	5.86%	HBsAg	Mumtaz *et al*. [37] 2002
	Northern Pakistan	ELISA	103858	3.3%	HBsAg	Khattak *et al*. [38] 2002
	Bahawalpur	LAT, ICT	345	5.64%	HBsAg	Fayyaz *et al*. [39] 2002

LAT: Latex agglutination test

**Table 3:** Percent prevalence rates of HBV infection in Healthcare workers, pregnant women and pediatric population.

Population type	Region	Methods	Population size	HBV positive (%)	HBV marker	Reference
**Health Care Personals**	Abbottabad	ELISA	125	2.4%	HBsAg	Sarwar *et al*. [41] 2008
	Muzaffarabad	RPHA, ELISA	199	4.1%	HBsAg	Naz *et al*. [42] 2002
**Pregnant women**	Karachi	ICT	2592	0.34%	HBsAg	Sheikh, [43] 2009
	Swat	ICT, ELISA	5607	3.98%	HBsAg	Khattak *et al*. [44] 2009
	Karachi	EIA	5902	4.6%	HBsAg	Sami *et al*. [45] 2009
	Lahore	ICT, ELISA	2439	2.2%	HBsAg	Batool *et al*. [46] 2008
	Bahawalpur	ICT, ELISA	300	12.3%	HBsAG, HBeAG, HBcAB, HBsAB,	Ahmad *et al*. [47] 2007
	Karachi	ICT, ELISA	25,482	1.57%	HBs Ag	Ali & Memon, [48] 2007
	Hyderabad	ICT, ELISA	103	12.6%	HBsAg	Yousfani *et al*. [49] 2006
	Rahim Yar Khan	ELISA	450	12.0%	HBsAg	Hakeem *et al*. [50] 2006
	Karachi	ICT, ELISA	245	3.26%	HBsAg	Mehnaz *et al*. [51] 2002
**Children**	Karachi	ELISA	3533	1.8%	HBsAg	Jafri *et al*. [52] 2006
	Lahore	RPHA, ELISA	392	2.04%.	HBsAg	Khan *et al*. [53] 1998

EIA: Enzyme Immunoassay, RPHA: Reverse Passive Hemagglutination Technique

**Table 4:** Percent prevalence of HBV infection in patients of different diseases in Pakistan.

Population type	Region	Methods	Population size	HBV positive(%)	HBV marker	Reference
**Surgical patients**	Karachi	EIA	496	5.0%	HBsAg	Moosa *et al*. [57] 2009
	Jacobabad Sindh	ICT	150	9.33%	HBsAg	Daudpota & Soomro, [58] 2008
	Karachi	ELISA	387	6.5%	HBsAg	Masood *et al*. [59] 2005
	Karachi	Latex method, ELISA	411	8.76%	HBsAg	Shirazi *et al*. [60] 2004
**Patients with cirrhosis**	Saidu Sharif, Swat	ELISA	110	21.81%	HBsAg	Khan *et al*. [62] 2009
	Dera Ismail Khan	ICT	60	46.67%	HBsAg	Mashud *et al*. [63] 2004
	Lahore	ICT, ELISA	94	23%	HBsAg, anti-HBcIgG, anti-HBs, and HBeAg	Khan *et al*. [64] 2002
	Lahore	ELISA	50	24%	HBsAg	Hussain *et al*. [65] 1998
**Patients with Hepatocellular carcinoma (HCC)**	Hyderabad	ELISA	200	21.0%	HBsAg	Ansari *et al*. [66] 2009
	Rawalpindi	ICT, ELISA	44	25%	anti-HBsAg, anti-HBcAb antiHBeAb	Mumtaz *et al*. [67] 2001
	Lahore	ELISA	30	20%	HBsAg	Kausar *et al*. [68] 1998
**Patients with hepatitis**	Rawalpindi	ICT	264	9.8%	HBsAg	Mumtaz & Aftab [69] 2005
	Hyderabad/Jamshoro	ELISA	100	41%	anti-HBs anti-HBc	Almani *et al*. [70] 2002
	Islamabad	ELISA	2574	15%	HBsAg	Tanwani & Ahmad [71] 2000
	Karachi	MEIA-Abbott	1225	2%	HBsAg	Mahmood [72] 2000
	Rawalpindi	RPHA or ELISA	4315	11.68%	HBsAg	Hussain & Ahmed [73] 1998
**Patients of Liver disease**	Karachi	ICT, ELISA, PCR	5193	32.6%	HBsAg	Ahmed *et al*. [74] 2010
	Peshawar	ICT, ELISA and PCR.	181	18.23%	HBs Ag or DNA	Khan [75] 2006
	Faisalabad	ELISA	100	29%	HBsAg	Bilal *et al*. [76] 2006
	Hazara Division	ICT	893	30.35%	HBsAg	Khan and Rizvi [77] 2003
**MTP (Thalassemic & Hemophiliac Children)**	Islamabad	ICT, ELISA	251	3.9%	HBsAg	Burki *et al*. [79] 2009
	Peshawar	ELISA	250	8.4%	HBsAg	Shah *et al*. [80] 2005
	Peshawar	ELISA	80	7.591	HBsAg	Mohammad *et al*. [81] 2003
	Peshawar	ELISA	40	5%	HBsAg	Hussain *et al*. [82] 2003
**Ophthalmic Patients**	Jamshoro/Hyderabad	ICT	931	4.6%	HBsAg	Junejo *et al*. [88] 2009
	Dera Ismail Khan	ICT, ELISA	1130	3.18%	HBsAg	Ahmad *et al*. [89] 2006
**IDU**	Peshawar	ELISA	250	22.4%	HBsAg	Alam *et al*. [86] 2007b
	Karachi	ELISA	161	7.5%	HBsAg	Altaf *et al*. [87] 2007

MTP: Multi-transfused Population, IDU: Injecting drug Users

**Table 5:** Summaries of the studies conducted on prevalence of HBV genotypes in Pakistan.

Authors	Region	Patients (n)	Genotype A	Genotype B	Genotype C	Genotype D	Genotype E	Genotype F	Untypable	Mixed
**Awan *et al*. **[96]** 2010**	All areas of Pakistan	300	43 (14.33%)	54 (18%)	83 (27.66%)	39 (13%)	2 (0.66%)	4 (1.33%)	31 (10.33%)	44 (14.66%)
**Ahmed *et al*. **[97]** 2009**	Punjab and Sindh	236	2 (0.85%)	-	14 (5.93%)	220 (93.22%)	-	-	-	-
**Baig *et al*. **[98]** 2009**	Karachi	315	65 (20%)	-	-	219 (70%)	-	-	-	31 (10%)
**Noorali *et al*. **[7]** 2008**	Karachi	180	-	-	-	150 (83.33%)	-	-	-	30 (16.66%)
**Hakim *et al*. **[8]** 2008**	Karachi	180	-	-	-	151 (83.89%)	-	-	-	29 (16.11%)
*Alam *et al*. [99]** 2007**	Patients from All four Provinces	110	5 (4.55)	27(24.54%)	-	66 (60%)	-	-	9 (8.18%)	3 (2.73%)
**Baig *et al*. **[100]** 2007**	Karachi	295	60 (20.34%)	-	-	208 (70.51%)	-	-	-	27 (9.15%)
**Alam *et al*. **[86]** 2007**	KPK	56	15 (8.92%)	-	-	35 (62.5%)	-	-	-	16 (28.57%)
**Abbas *et al*. **[101]** 2006**	Karachi	109	-	-	-	109 (100%)	-	-	-	-
**Idrees *et al*. **[1]** 2004**	Patients from All four Provinces	112	24 (21.42%)	20 (17.86%)	46 (41.07%)	9 (8.03%)	-	-	5 (4.46%)	8 (7.14%)
**Total**		1893	190 (10.03%)	101 (5.335%)	143 (7.55%)	1206 (63.71%)	2 (0.105%)	4 (0.21%)	45 (2.377%)	188 (9.931%)

KPK: Khyber Pakhtunkhwa Province

*The percentage values are different in the original manuscript as the authors considered the untypable samples as negative for genotype.

Formula used for determination of mean prevalence in each population group

$$\mu =(\Sigma {\text{ x}}_{\text{i}})/\text{N}$$

Formula used for determination of standard deviation (SD) in each population group

$$\sigma =\sqrt{\frac{1}{N}\sum_{i=1}^{N}{\left(x_{i}-\mu \right)}^{2}}.$$

Where "x" is the percent HBV prevalence reported in each study and "N" is the total number of studies in the population groups.

## HBV prevalence in various population groups

### General population (healthy population)

Eleven different studies reported the percent prevalence rates of hepatitis B virus of in general population as 4.3318% ± 1.644% (2.46%-8.06%) [6-8,10-17], while six different studies involving healthy recruits showed the prevalence rate of 4.276% ± 1.646%. [18-23]. HBV prevalence of 5.75% ± 0.212% was observed in prisoners [24,25]. Fourteen different studies showed the prevalence rate of 3.93% ± 1.58% in healthy blood donors in Pakistan [26-39]. HBV prevalence of 9.0% has been reported in professional blood donors [40]. Two different studies showed the prevalence of HBV in health care workers as 3.25% ± 1.202% [41,42]. Nine studies showed the HBV prevalence of 5.872% ± 4.984% in pregnant women [43-51], while two different studies demonstrated 1.92% ± 0.169% prevalence in children [52,53]. A very high frequency of ≥12% HBV infection in pregnant females has been reported in Bahawalpur, Hyderabad and Rahim Yar Khan regions [47,49,50]. Up to 21% of the children born of HBV infected females were infected [47], while Kazmi *et al*. [54] showed a high prevalence of 90% in children born of HBV positive mothers. Quddus *et al*. [55] showed HBV prevalence of 8.3% in Afghan refuges residing in Pakistan. Anwar *et al*. [56] showed a high prevalence rate of HBV that was 11.65% in female prostitutes in Lahore, Pakistan. Frequency of viral hepatitis in blood donors is higher in Bahawalpur as compared to rest of the world [33].

These studies show that Southern Punjab, Interior Sindh, District Tatta, Kurrum agency and some areas of Lahore have very high HBV prevalence of >5%, hence mass vaccination and awareness programs in these areas on urgent basis is suggested.

### Surgical patients

Four different studies showed 7.397% ± 2.012% HBV prevalence rates in patients undergoing surgery [57-60]. The lack of routine serological screening in Pakistani hospitals prior to surgery is one of the factors responsible for increased disease transmission [59]. It is recommended that every case undergoing surgery should be screened for hepatitis B and C virus infections [61].

### Patients with hepatitis, liver diseases, HCC and cirrhosis

Four different studies showed the percent HBV prevalence of 28.87% ± 11.90% in patients with cirrhosis [62-65] while 22% ± 2.645% HBV prevalence was shown by three different studies in patients with HCC [66-68]. Five studies in patients with hepatitis showed the percent prevalence of 15.896% ± 14.824% [69-73] while four different studies in patients with different liver diseases showed the prevalence of HBV as 27.54% ± 6.385% [74-77].

### Muti-tranfused population (thalassemic and hemophilic patients)

Thalassemic and hemophilic patients require life-long blood transfusions, so it is necessary to obtain screened blood from a reputable source, because the multitransfused population is more prone to blood-borne pathogens [78]. Four different reports showed percent HBV infection of 6.223% ± 2.121% in multi-tranfused population [79-82].

### Intravenous drug users (IDU)

Pakistan is estimated to have 4 to 4.8 million drug users with 180,000 IDUs [83]. Strathdee *et al*. [84] observed significant increase in needle sharing in IDUs since 2001. Among them Afghan refugees have higher levels of needle sharing as compared to the local IDUs [85]. Two different reports showed a very high HBV prevalence of 14.95% ± 10.536% in injection drug users [86,87].

### Patients with other diseases

Two different reports show the prevalence of HBV in ophthalmic patients to be 3.89% ± 1.004% [88,89], percent prevalence of HBV was 12.4% in patients on hemodialysis [90], 26% in psychiatric patients [91], 10.2% in patients advised for liver function tests [92], 1.5% in patients with dermatoses caused by *lichen planus *[93] and 2.02% in orthopedics patients [94]. High prevalence among psychiatric patients could be due to razor sharing, facial and armpit shaving from barbers and carelessness during injuries.

### Genotypes

Hepatitis B virus exists in eight different genotypes (A-H) and its prevalence differs with differs by geography and ethnicity [95]. Ten different studies (Table 5) conducted at different regions of Pakistan showed that the most prevalent HBV genotype in Pakistan is genotype D with overall prevalence rate of 63.71% followed by genotype A (10.036%), genotype C (7.55%) and genotype B (5.335%) while untypable and mixed genotypes were 2.377% and 9.931%, respectively [1,7,8,87,97-102]. The most detailed study recently conducted by Awan *et al*. [96] showed that the most emerging genotype in Pakistani population is genotype C with the prevalence rate of 27.66%, which is a bad news as it is more common in cirrhotic patients and is known to be associated with more severe liver diseases. Moreover, Previous studies also shows that genotype D have more severe disease, less responsive to interferon therapy as compared to genotype A and B and have higher HBV DNA levels. This genotype also has specific viral sequence patterns that may predict long-term response to lamivudine treatment [101]. However, further studies are needed to characterize prevalence of different genotypes, their relative severity and treatment response rates in Pakistani population.

### Risk factors associated with HBV infection

History of dialysis for more than 2 years is a risk factor for dialysis patients [90]. Major risk factors for mother to infant transmission include increasing maternal age, number of pregnancies, repeated injections and addiction [51]; major risk factors in surgical patients include re-use of contaminated syringes, contaminated surgical instruments and blood products [59]; risk factors in pregnant women (antenatal) include ear and nose prick, history of jaundice among them or with their partner [49], history of blood transfusions, history of injections [46,49], tooth extraction [46]; in prisoners significant risk factors were intravenous drug abuse [24,25], rural origin and shaved by barber [25]; in orthopedic patients common risk factors are previous history of surgery or blood transfusion [94]. Major risk factors for health care workers are dental procedures, needle prick and surgical procedures [41]; most important risk factors for HBV infection in young *recruits *were sharing of razors, history of intravenous injections, jaundice in the subject and jaundice in family [22]; in children key risk factors were injection in the past, surgical and dental procedures, blood transfusion, accidental cuts at barber shops and umbilical cord cut through unsterilized instruments at home [102]; in thalassaemic children there is a definite risk factor of repeated blood transfusion [79], while main risk factors among the obstetrical and gynecological population were unsafe surgery, injections and inadequately screened blood transfusions [45]. Qureshi *et al*. [103] compared male patients suffering from chronic hepatitis with healthy people as control and found very strong relationship of the HBV infection with history of dental treatment, surgery and history of taking injections. It is established fact that HBsAg does not cross the placental barrier however; the infection in children/newborns may occur at the time of birth or soon after birth. The infants of HBV positive mothers must be vaccinated soon after birth and HBIG immediately within 24 hours of birth [54]. Relatively low prevalence in the female prostitutes (high risk group) reflects the effect of legal, social and religious constraints in Pakistani society [56]. Many of the Afghan refuges (most of them are children) are rag pickers who collect used syringes and needles dumped outside the hospitals and hence are one of the most vulnerable groups to viral hepatitis in Pakistan [104]. Most of these factors are easily preventable and need awareness in general population and the healthcare workers.

Preventive strategies for HBV infection include healthy blood transfusion services along with safe sex [34], vaccination against HBV [40,103], shaving by barbers needs to be discouraged [103] and better training of healthcare workers [105]. Paid blood donation should be prohibited [85]. All patients needing surgery should be screened for viral hepatitis and there should be separate operation theatres facilities for these patients [94]. In addition reuse of razors in many barber shops that may spread hepatitis in a substantial number of individuals [106] and must be discouraged. Importantly, the most common risk factors and modes of HBV transmission in this country differ in importance in various community groups [23].

In Turkish population, HLA-A24 and Cw1 has been associated with low risk for HBV-related chronic liver disease and HLA- B13, B8, DR7, DR13 and DQ3 were found associated with high risk for chronic HBV infection [107]. However, there is no study that describes cellular or molecular mechanism of HBV infection in Pakistani population.

### Awareness about HBV in Pakistani population

Nasim *et al*. [108] conducted a questioner bases survey in Karachi to assess knowledge about viral hepatitis among college girls and showed that 57% of them do not have information about transmission of hepatitis B virus. In another survey [109] at obstetric and gynecology clinic at Hyderabad showed that most of the women (67.76%) know that HBV is a viral disease, 75.20% responded that it affect liver, 33.88% believed that it could be transmitted by infected blood transfusion, 17.35% believed that it could be transmitted from mother to child, 19.0% mentioned sexual intercourse responsible for HBV infection, while 40.49% and 38.0% mentioned contaminated needles and un-sterilised instruments as a source of HBV infection, respectively. Mengal *et al*. [110] surveyed nursing students at nursing school, Bolan medical complex hospital, Quetta and reported that only 37.2% of them were completely vaccinated and 25.0% had not been vaccinated for HBV. Chaudhry *et al*. [111] repoted that 97.4% of the barbers at Islamabad use new blade for every customer but only 38% of them has knowledge about routes of infection of HBV and HCV. In another study, Waheed *et al*. [112] reported that 39.6% of the barbers at Rawalpindi and Islamabad knew that Hepatitis B and hepatitis C were viral diseases, 90.7% thought that hepatitis could spread by blade sharing, 26.6% knew that it can lead to cancer and 47.8% knew that a vaccine for HBV was available. Ali *et al*. [113] reported that 78.8% of the rural population of Faisalabad was unaware of viral hepatitis. In another study, Asif *et al*. [114] reported that only 17.6% of the rural population of Nowshera was aware of the fact that Hepatitis B and C are transmitted by a virus. Talpur *et al*. [115] stated that there is significant lack of knowledge and poor attitude towards HBV and HCV in surgical patients at Nawabshah area. We suggest aggressive public awareness programs especially in rural areas and people at high risk to decrease the burden of HBV infection in Pakistan.

## Conclusions

This article reviews prevalence of HBV in different areas and population groups in Pakistan, along with awareness status, risk factors and genotypes in Pakistani population. Prevalence of HBV infection varies with population residing in different regions of Pakistan. The present literature shows that Afghan refuges in Pakistan, IDUs, professional blood donors, health care professionals, prisoners, multiple transfused patients, patients with HCC, psychiatric patients, general population of some specific areas like Southern Punjab, Interior Sindh, District Tatta, Kurrum agency, Baltistan and some areas of Lahore have very high HBV prevalence of more than 5%, and there is urgent need of mass vaccination and awareness programs. Further studies are needed to characterize HBV prevalent in Pakistan at molecular level. Moreover, both host and viral factors associated with molecular and cellular mechanism of HBV infection in Pakistani population needs to be explored.

## Competing interests

The authors declare that they have no competing interests.

## Authors' contributions

MA and MI conceived the study and designed the inclusion criteria. MA searched the literature and drafted the manuscript. MI, IR and AH critically reviewed the manuscript. IR, SS, SA, SB and LA helped MA in literature search, data extraction and statistical analysis. All the authors read and approved the final manuscript.
